# Homology to peptide pattern for annotation of carbohydrate-active enzymes and prediction of function

**DOI:** 10.1186/s12859-017-1625-9

**Published:** 2017-04-12

**Authors:** P. K. Busk, B. Pilgaard, M. J. Lezyk, A. S. Meyer, L. Lange

**Affiliations:** Department of Chemical and Biochemical Engineering, Technical University of Denmark, Søltofts Plads, Building 229, 2800 Kgs. Lyngby, Denmark

**Keywords:** Carbohydrate-active enzymes, Genomics, Annotation, Software

## Abstract

**Background:**

Carbohydrate-active enzymes are found in all organisms and participate in key biological processes. These enzymes are classified in 274 families in the CAZy database but the sequence diversity within each family makes it a major task to identify new family members and to provide basis for prediction of enzyme function. A fast and reliable method for *de novo* annotation of genes encoding carbohydrate-active enzymes is to identify conserved peptides in the curated enzyme families followed by matching of the conserved peptides to the sequence of interest as demonstrated for the glycosyl hydrolase and the lytic polysaccharide monooxygenase families. This approach not only assigns the enzymes to families but also provides functional prediction of the enzymes with high accuracy.

**Results:**

We identified conserved peptides for all enzyme families in the CAZy database with Peptide Pattern Recognition. The conserved peptides were matched to protein sequence for *de novo* annotation and functional prediction of carbohydrate-active enzymes with the Hotpep method. Annotation of protein sequences from 12 bacterial and 16 fungal genomes to families with Hotpep had an accuracy of 0.84 (measured as F1-score) compared to semiautomatic annotation by the CAZy database whereas the dbCAN HMM-based method had an accuracy of 0.77 with optimized parameters. Furthermore, Hotpep provided a functional prediction with 86% accuracy for the annotated genes. Hotpep is available as a stand-alone application for MS Windows.

**Conclusions:**

Hotpep is a state-of-the-art method for automatic annotation and functional prediction of carbohydrate-active enzymes.

**Electronic supplementary material:**

The online version of this article (doi:10.1186/s12859-017-1625-9) contains supplementary material, which is available to authorized users.

## Background

Carbohydrate-active enzymes are produced by all organisms to accomplish enzymatic modification of carbohydrate-containing compound both intra- and extracellularly. Hence, this enzyme group is relevant for understanding central biological processes such as sugar metabolism, protein glycosylation and, on an ecological level, for global biomass synthesis and degradation. It is not surprising that carbohydrate-active enzymes are used in medical and industrial biotechnology. The CAZy database (http://www.cazy.org/) was founded in 1991 and contains a unique classification of carbohydrate-active enzymes including carefully curated information about enzyme sequence, structure and function [1]. Currently, the publicly available information in the CAZy database consists of almost 400.000 unique protein sequences classified in more than 300 families.

Despite the abundant information in the CAZy database, *de novo* annotation of carbohydrate-active enzymes is not a trivial task. State-of-the-art methods involve automatic identification by matching the sequences of interest to protein models generated directly from sequences in the CAZy database or indirectly from protein domain models from other databases or by BLAST search followed by manual curation of the data [1–4].

Entirely automatic annotation methods have been developed based on hidden Markov model (HMM) recognition of all or a subset of the enzymes in the CAZy database and are available as web-based services [5–7]. E.g., the dbCAN method was made by refining HMM models from the Conserved Domain Database to fit the families in the CAZy database and supplementing the database with new HMM models for the families in the CAZy database that are not modelled in the Conserved Domain Database [7].

Even when it is possible to annotate a protein to a specific family this does not necessarily allow an exact prediction of its enzymatic activity. This is due to that the classification of the carbohydrate-active enzymes in the CAZy database is based on protein sequence and structure similarity [1]. Thus, in many cases the classification does not reflect enzymatic activity [1]. Hence, proteins with identical enzymatic activity are classified in different families and most of the families contain proteins with different enzymatic activities.

Identification of short, conserved motifs can be used to group related protein sequences and will often pinpoint proteins with the same enzymatic activity [8, 9]. Furthermore, the method Homology to Peptide Pattern (Hotpep) matches the short, conserved motifs to undescribed protein sequences to obtain a fast, sensitive and precise annotation of carbohydrate-active enzymes to families [10]. Moreover, when experimental data on enzymatic activity is available Hotpep allows prediction of the enzymatic activity of the proteins. In practice, the experimental data on enzyme activity collected in the CAZy database can be used to predict the enzymatic activity of approximately 75% of the carbohydrate-active enzymes in a genome with 80% accuracy [9, 10].

We used the method Peptide Pattern Recognition (PPR) to identify short, conserved sequence motifs for all enzyme families in the CAZy database. The peptide patterns were combined with Hotpep to obtain a stand-alone software for automatic annotation and functional prediction of carbohydrate-active enzymes. As an example, to illustrate the workability of the approach, annotation of protein sequences from 12 bacterial and 16 fungal genomes was addressed. Hotpep had an F1 score of 0.86 (sensitivity = 0.88, precision = 0.84) for predicting carbohydrate-active enzymes in 12 bacterial genomes and an F1 score of 0.82 (sensitivity = 0.77, precision = 0.88) for predicting carbohydrate-active enzymes in 16 fungal genomes compared to semiautomatic annotation by the CAZy database tools for carbohydrate-active enzyme annotation [1, 4]. Moreover, Hotpep correctly predicted the activity of 86% of the characterized carbohydrate-active enzymes in the CAZy database.

The carbohydrate binding modules (CBM) are not defined as carbohydrate-active enzymes *per se* but are carbohydrate binding domains within multidomain carbohydrate-active enzymes [11]. Using short, conserved peptides for the CBM families in the CAZy database Hotpep annotates the CBMs with an F1 score of 0.87.

The Hotpep stand-alone application is available for download from Sourceforge for use on desktop computers with the MS Windows operative system.

## Implementation

**Fig. 1:**
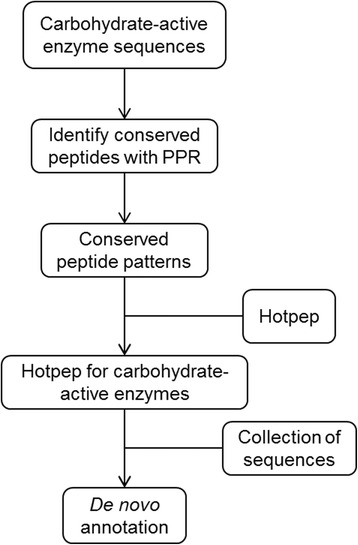
Steps in development and use of Hotpep for Carbohydrate-active enzymes

Development and testing of Hotpep for carbohydrate-active enzymes followed a number of steps as outlined (Fig. 1).

### Protein sequences

The first step was to download sequences for all members of each carbohydrate-active enzyme family in the CAZy database (www.cazy.org [1]) from Genbank (https://www.ncbi.nlm.nih.gov/ [12]) in August, 2016. The CBM families were downloaded in February, 2017. Sequences that were 100% redundant or 100% identical to a part of another sequence were removed.

### Identification of short, conserved peptides

PPR was used for identification of short, conserved peptides in each family of carbohydrate-active enzymes as previously described [9, 10, 13]. Briefly, for each family PPR found the largest group of proteins that contained at least 10 of 70 conserved hexamer peptides. The length of the conserved peptides (hexamers), the number of conserved peptides per protein (10) and the total number of conserved peptides per group (70) were chosen as they were the conditions that gave the best rate of prediction of protein function in empirical testing of peptide lengths from trimers to decamers, 5 – 40 conserved peptides per protein and 30 – 200 conserved peptides per group [9]. Moreover, the minimum frequency of each conserved peptide in a group was 0.20 as this threshold gives the best rate of prediction of protein function [9]. For CBM domains the parameters 30 conserved hexapeptides per PPR group and 3 conserved peptides per protein were used for PPR analysis.

The first group of proteins identified by this method was named group 1. Next, PPR found the second largest group of proteins, not including any proteins from group 1. This group of proteins was named group 2 and so on. The analysis was stopped when less than five proteins were grouped together.

In this way a number of groups consisting of a list of protein sequences and a list of conserved peptides were generated for each family in the CAZy database. Groups including proteins with a described enzyme activity as reported in the CAZy database were assigned the same function as the enzymes as previously described [9].

For AA families 9, 10 and 11 the conserved peptide lists of the previously described expanded families were used [13].

### Sequence collections

Genome-annotated protein products (“*_protein.faa.gz” files) were downloaded from Genbank for 12 bacterial (Table 1) and 16 fungal species (Table 2). For comparison of annotation from genomes and from predicted proteins the files *_genomic.fna.gz (genome assembly) and *_protein.faa.gz (protein products annotated on the genome assembly) for the following fungi *Thermothelomyces thermophile* (Accession: GCF_000226095.1), *Talaromyces stipitatus* (Accession: GCA_000003125.1), *Botryobasidium botryosum* (Accession: GCA_000697705.1), *Coprinopsis cinerea* (Accession: GCA_000182895.1), *Serendipita indica* (Accession: GCA_000313545.1), *Mucor circinelloides* (Accession: GCA_000401635.1) and *Rhizopus delemar* (Accession: GCA_000149305.1) were downloaded from Genbank.

**Table 1:** Bacterial strains and accession numbers

Name	Phylum	Isolated from	Accession numbers
*Bacteroides cellulosilyticus WH2*	Bacteroidetes	Gut and stomach	GCA_000463315.1
*Caldicellulosiruptor saccharolyticus DSM8903*	Firmicutes	Wood Thermophilic anaerobe	GCA_000016545.1
*Deinococcus peraridilitoris DSM19664*	Deinococcus-Thermus	Coastal desert	GCA_000317835.1
*Desulfotomaculum gibsoniae DSM7213*	Firmicutes	Freshwater ditch	GCA_000233715.3
*Enterobacter lignolyticus SCF1*	Proteobacteria	Tropical forest soil	GCA_000164865.1
*Melioribacter roseus P3M-2*	Ignavibacteriae	Wooden surface of a chute	GCA_000279145.1
*Prevotella ruminicola 23*	Bacteroidetes	Gut	GCA_000025925.1
*Rhodococcus jostii RHA1*	Actinobacteria	Hexachlorocyclohexane-contaminated soil	GCA_000014565.1
*Ruminiclostridium thermocellum ATCC27405*	Firmicutes	Soil/manure	GCA_000015865.1
*Teredinibacter turnerae T7901*	Proteobacteria	Intracellular in shipworm	GCA_000023025.1
*Thermacetogenium phaeum DSM12270*	Firmicutes	thermophilic anaerobic methanogenic reactor	GCA_000305935.1
*Thermoanaerobacterium thermosaccharolyticum DSM571*	Firmicutes	Soil	GCA_000145615.1

**Table 2:** Fungal strains (basidiomycotae) and accession numbers

Name	Order	Life style	Accession numbers
*Postia placenta*	*Polyporales*	Brown rot	GCA_000006255.1
*Fomitopsis pinicola*	*Polyporales*	Brown rot	GCA_000344655.2
*Gloeophyllum trabeum*	*Gloeophyllales*	Brown rot	GCA_000344685.1
*Coniophora puteana*	*Boletales*	Brown rot	GCA_000271625.1
*Dacryopinax sp.*	*Dacrymycetales*	Brown rot	GCA_000292625.1
*Tremella mesenterica*	*Tremellales*	Mycoparasite	GCA_000271645.1
*Dichomitus squalens*	*Polyporales*	White rot	GCA_000275845.1
*Trametes versicolor*	*Polyporales*	White rot	GCA_000271585.1
*Fomitiporia mediterranea*	*Hymenochaetales*	White rot	GCA_000271605.1
*Auricularia delicata*	*Auriculariales*	White rot	GCA_000265015.1
*Punctularia strigosozonata*	*Corticiales*	White rot	GCA_000264995.1
*Heterobasidion annosum*	*Russulales*	White rot	GCA_000320585.2
*Stereum hirsutum*	*Russulales*	White rot	GCA_000264905.1
*Phanerochaete_carnosa*	*Polyporales*	White rot	GCA_000300595.1
*Ceriporiopsis subvermispora*	*Polyporales*	White rot	GCA_000320605.2
*Phlebiopsis gigantea*	*Polyporales*	White rot	GCA_000832265.1

### Annotation with Hotpep

Genomic fragments were annotated as previously described [10]. Annotation of protein products from genome assemblies was performed on full-length predicted protein sequences essentially as described [9]. Briefly, protein sequence was given a score for each group-specific peptide lists for each family by:

1. Finding all the conserved peptides from the list that were present in the sequence.
2. Sum the frequency of these peptides to obtain the group-specific frequency score.

A hit was considered significant if the protein sequence:

1. Included three or more conserved peptides from a group.
2. The frequency score for the peptides was higher than 1.0
3. The conserved peptides represented at least ten amino acids of the protein sequence.

If a protein satisfied all three conditions it was assigned to the family and to the PPR group with the highest group-specific frequency score. Moreover, if this group had been assigned a function by the PPR analysis, the same function was predicted for the protein [9].

Hotpep including the conserved peptide patterns described here is available for download as an application for the MS Office operative system from Sourceforge.

### Annotation with dbCAN

The protein products from each genome were annotated *de novo* with the dbCAN web service for protein annotation with standard parameters and with optimized parameters (E-value < 10^−18^; coverage > 0.35 for bacteria and E-value < 10^−17^; coverage > 0.45 for fungi) by downloading scripts and HMMs as described (http://csbl.bmb.uga.edu/dbCAN/annotate.php, [7]).

### Statistical analysis

The following values were calculated for pairwise comparison of two annotation methods:

True positives = Number of hits found by both screening methods. False positives = Number of proteins found by the screening method being tested but not by the reference method. False negatives = Number of proteins found by the reference method but not by the screening method being tested.

Sensitivity was calculated as True positives/(True positives + False negatives); Precision (positive prediction value) was calculated as True positives/(True positives + False positives) and F1 score (the harmonic mean of precision and sensitivity) was calculated as (2 × True positives)/(2 × True positives + False positives + False negatives).

## Results and discussion

Short, conserved peptides identified in the carbohydrate-active enzyme from the glycoside hydrolase families in the CAZy database can be used for fast, efficient and reliable approach for annotation by the Hotpep method [10]. Moreover, groups of carbohydrate-active proteins sharing the same short, conserved peptides do often have the same enzymatic activity [9]. Thus, by comparing the rich information on experimentally characterized enzymes in the CAZy database with the PPR grouping of the enzymes it is possible to predict the enzymatic activity of the uncharacterized members of the groups with 80% accuracy. In this way, a functional prediction was obtained for 72% of the annotated glycoside hydrolases in 39 fungal genomes [10].

To accomplish automatic annotation of all carbohydrate-active enzymes with Hotpep we downloaded all sequences in the families of the five enzyme classes: Carbohydrate esterases (CE), Glycoside hydrolases (GH), Auxiliary activities (AA), Polysaccharide lyases (PL) and Glycosyl transferases (GT). A total of 594,121 accession numbers were found in the CAZy database and reduced to 380,269 non-redundant protein sequences before each family was sorted into groups of proteins sharing up to 70 short, conserved hexapeptides and assignment of function to each group containing more than two functionally characterized members (Additional file 1). In total 36% of the 5590 PPR groups for all enzyme families included functionally characterized proteins. These groups with associated functions contained 65% of the PPR-grouped proteins. For the glycoside hydrolases, 41% of the groups included functionally characterized proteins and a total of 74% of all proteins, in agreement with the previous report of a functional prediction of 72% of the glycoside hydrolases [10].

For the CBM class of carbohydrate-binding modules we found 71,253 accession numbers in the CAZy database resulting in 45,048 non-redundant protein sequences. Due to the short length of most CBM domains [7, 11] it was uncertain whether the standard parameters of 70 conserved peptides per PPR group and 10 conserved peptides per protein were optimal for annotation of CBMs. Therefore, different parameters for PPR were tested for classification of the isolated CBM domains followed by Hotpep annotation of the full-length proteins and comparison to the annotation in the CAZy database. There was little variation in the F1 score (0.83 - 0.87) within the range of tested parameters (Additional file 2) in agreement with the notion that PPR groups are fairly stable within a large range of parameters [9]. The parameters 30 conserved peptides per PPR group and 3 conserved peptides per protein gave the highest F1 score of 0.87 and were chosen for annotation of CBMs.

Hotpep annotates proteins by matching the lists of conserved peptides of a group to the protein sequences of interest [10, 13, 14]. Any sequence that fulfills a number of criteria (see Implementation) of which the most important is that the sequence should include at least three of the conserved peptides, will be annotated to the protein group. We combined Hotpep with the lists of conserved peptides for all enzyme families in the CAZy database to an application that can identify members of all carbohydrate-active enzyme families and CBMs. The AA9, AA10 and AA11 conserved peptides were substituted with the AA9exp, AA10exp and AA11exp conserved peptides that represent a more complete description of the sequence variation in these families [13]. The complete lists of peptides and frequencies are available for download at Sourceforge together with the accession numbers of the sequences for each group and the library of EC functional scores for each group.

**Fig. 2:**
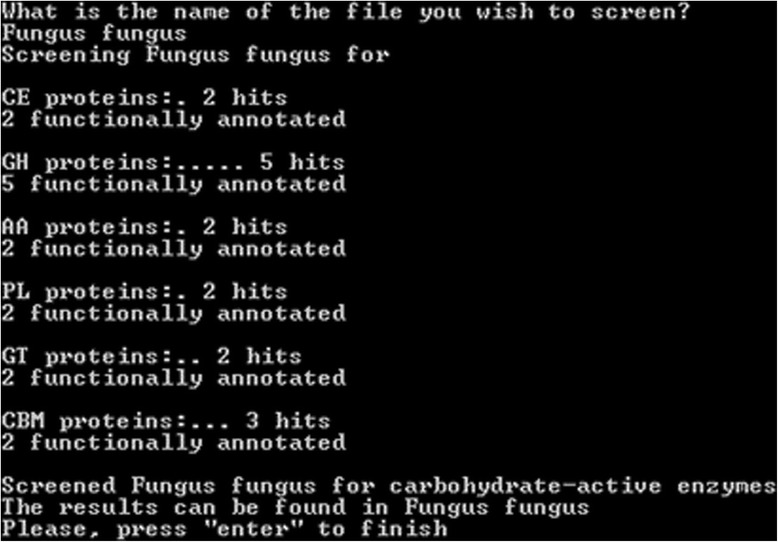
Hotpep user interface. Double-clicking on the Hotpep icon opens a DOS promt where the name of the sequence directory (e.g., “Fungus fungus”) is entered

The input for annotation with Hotpep is a text file with predicted protein sequences in fasta format. The algorithm is started by double-clicking the Hotpep icon. This will open a DOS prompt, where the user writes the name of the input file containing the fasta-formatted protein sequences (Fig. 2).

**Fig. 3:**
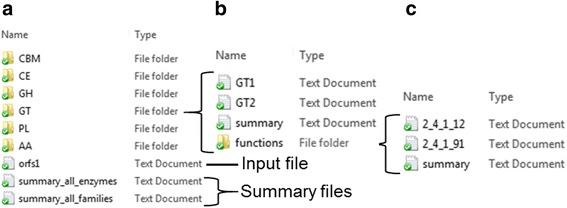
Organization of the Hotpep output. **a**. The output is delivered in the sequence directory with one directory for each enzyme class in the CAZy database, a file containing a summary of the results and a file with all the families found for each accession number. **b**. Each of the class directories contains files with the hits for each family, a summary and a directory with functional predictions. **c**. The folder with functional predictions contains files for each EC number found and a summary

Hotpep screens the input sequences for members of all families in the CAZy database. This will take 5 – 20 min for all predicted genes in a bacterial or fungal genome. Several genomes can be annotated in parallel by running Hotpep several times. The results files are saved in six directories, one for each class of carbohydrate-active enzymes, one for the CBMs and two summary files: One with the number of hits for each family and one with the accession number of each hit and the families annotated for this hit (Fig. 3a). The latter file gives an overview of the number and families for multidomain enzymes.

**Fig. 4:**
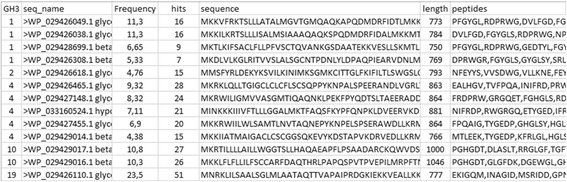
Hotpep output. An output files with hits for the GH3 family opened in MS Excel. The columns (from left to right) contain the group where the sequence is annotated, the name of the sequence, the sum of the frequency of the conserved peptides, the number of conserved peptides, the protein sequence, length of the sequence and the sequences of the conserved peptides

**Fig. 5:**
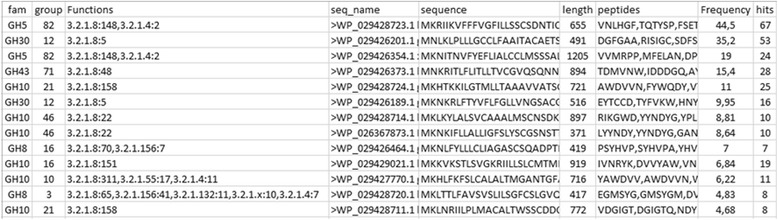
Hotpep output for functional prediction. Same as Fig. 4 with the addition of a column labelled “Functions” with information on the putative functions of the annotated sequence

The results for each enzyme class is a number of text files (Fig. 3b) prepared for import into MS Excel, LibreOffice or similar spreadsheet applications (Fig. 4). The columns in the spread sheet designates the group where the sequence is annotated, the name of the sequence, the sum of the frequencies of the conserved peptides [10, 14], the number of conserved peptides, the protein sequence, length of the sequence and the sequences of the conserved peptides. In addition, the directories contain a subdirectory with files including prediction of the activities of the enzymes arranged according to EC class (Fig. 3c). As the CBMs are binding modules associated to enzyme domains the predicted function is often the predicted function of the associated enzyme domain as described in the CAZy database. The files with functional prediction contain a column with the prediction of the enzymatic function according to EC class (Fig. 5). The information in this column consists of one or more EC numbers each followed by a colon and a number designating the sum of the number of conserved peptides in each characterized protein in the group. The higher this number, the more proteins in the group have the enzymatic activity represented by the EC number. E.g.; in family GH43 group 71 there are 48 conserved peptide matches to enzymes characterized as endoxylanases (EC 3.2.1.8) (Fig. 5). For family GH8 group 3 there are 65 conserved peptide matches to enzymes characterized as endoxylanases (EC 3.2.1.8) but also 41 conserved peptide matches to enzymes characterized as exo-oligoxylanase (EC 3.2.1.156) in addition to matches to enzymes with other activities (Fig. 5). Hence, expression and enzymatic characterization of the sequence with the accession number WP_029428720.1 annotated to this group is necessary to decide whether it is an endoxylanase or an exo-oligoxylanase as the scores for these two activities are similar.

This method correctly predicts 80 – 95% of enzyme activities [9, 10]. To test this further, we used Hotpep to predict the function of 8812 experimentally characterized carbohydrate-active enzymes (Additional file 3). Hotpep correctly predicted the function of 86% of the enzymes. This result supports the previous finding that proteins sharing conserved peptides often but not always have the same activity [9]. Hence, enzymatic activities for individual sequences predicted by Hotpep should be used as a guideline for functional characterization. In an analysis of annotation of glycosyl hydrolases from ORFs in genome fragments with Hotpep it was found that the glycosyl hydrolases that were overlooked by Hotpep could be detected when the full-length amino acid sequence of the enzymes were used for annotation [10]. This finding suggests that more true positive hits are obtained by examining full-length coding regions rather than ORFs containing single exons. To test this notion we compared the annotation of all carbohydrate-active enzymes in seven fungal genomes to annotation of predicted proteins from the same genomes. The fungi were selected to include genome assemblies and predicted proteins from different research groups to avoid methodical bias. The results showed that 31% more carbohydrate-active enzymes were found by annotation of the predicted proteins from the genomes compared to annotation of ORFs in fragments of the genomes (Additional file 4) in agreement with the previous report [10]. Hence, although exon-intron structure of eukaryotic genes makes them difficult to predict [15] a higher sensitivity in prediction of carbohydrate-active enzymes is obtained by annotating from predicted proteins rather than from ORFs in genome fragments.

Annotation with Hotpep of predicted proteins from 12 bacterial genomes was compared to state-of-the-art semiautomatic annotation reported in the CAZy database [1]. The selected genomes were from bacteria with different lifestyles including bacteria known to degrade extracellular carbohydrates.

The CAZy database reported slightly less carbohydrate-active enzymes than Hotpep for the 12 bacterial genomes (Table 3). We have previously found that Hotpep annotation of fungal genomes are largely in agreement with the results reported in the CAZy database and that the differences between the annotations may be due to genes overlooked by either Hotpep or in the CAZy database [10]. This is a natural effect of the fact that the families in the CAZy database are growing as new members are discovered and some of the families are redefined [1]. E.g.; the lytic polysaccharide monooxygenases (LPMOs) originally classified in the GH61 and CBM33 families [9, 16, 17] were later reclassified to the AA9, AA10 and AA11 families [18, 19]. In view of this plasticity of the CAZy database it is difficult to precisely determine the correct annotation of carbohydrate-active enzymes in a given dataset [7]. However, if the annotation reported in the CAZy database is defined as correct, then it means that the Hotpep annotation has a sensitivity of 0.88 and a precision of 0.84 (Table 3). This gives an F1 score of 0.86, which means that the methods on average agree on 86% of the number of predicted carbohydrate-active enzymes.

**Table 3:** Annotation of 12 bacterial genomes

Method	CAZy^a^	Hotpep	dbCAN web	dbCAN download
Annotated proteins	1768	1839	2300	1749
True positives	-	1546	1701	1571
False positives	-	296	599	178
False negatives	-	220	67	197
Sensitivity	-	0.88	0.87	0.89
Precision	-	0.84	0.71	0.90
F1 score	-	0.86	0.84	0.89

^a^ www.cazy.org

It was reported that automatic identification with the HMM signatures in dbCAN is a highly precise and sensitive method for annotation of carbohydrate-active enzymes [7]. Annotation of the 12 bacterial genomes with the dbCAN web service (http://csbl.bmb.uga.edu/dbCAN/annotate.php) gave a higher number of hits than the annotation in the CAZy database resulting in a sensitivity similar to Hotpep but with lower precision and F1 score (Table 3). However, annotation of the 12 bacterial genomes with the downloaded dbCAN HMMs and optimized parameters [7] gave a lower number of hits than the annotation in the CAZy database resulting in slightly higher sensitivity, precision and F1 score than Hotpep (Table 3). Thus, although the downloadable dbCAN is more difficult to use than the web service as the user has to both download the dbCAN HMMs and install the HMMER 3.0 package [7] the extra effort pays of in the form of a more accurate annotation. In summary, the comparison of the annotation methods showed that the CAZy database, Hotpep and downloaded dbCAN were most in agreement whereas the dbCAN web service annotates a higher number of genes as encoding carbohydrate-active enzymes.

To assess the performance of Hotpep for identification of eukaryotic genes, 16 fungal genomes that have been sequenced and annotated by The Joint Genome Institute and the CAZy database tools by Hori et al. [4] were selected for annotation. Testing on these genomes has the benefit that many of the carbohydrate-active enzymes from these fungi are not part of the CAZy database and has thus not been part of the dataset used to make the conserved peptide patterns used by Hotpep.

In case of the fungal genomes, Hori et al. [4] found slightly more carbohydrate-active enzymes than Hotpep (Table 4). However, Hotpep had an F1 score of 0.82 relative to the annotation by Hori et al., whereas annotation with dbCAN web service and downloaded dbCAN with optimized parameters only had F1 scores of 0.68 and 0.72, respectively (Table 4). Hence, for annotation of the fungal genes Hotpep and Hori et al. gave the most similar result whereas the dbCAN web service and the downloaded dbCAN predicted a higher number of carbohydrate-active enzymes. Summarizing the results for prediction of bacterial and fungal genes Hotpep had a combined F1 score of 0.84, dbCAN web service had an F1 score of 0.75 and downloaded dbCAN with optimized parameters had an F1 score of 0.77.

**Table 4:** Annotation of 16 fungal genomes

Method	JGI/CAZy^a^	Hotpep	dbCAN web	dbCAN download
Annotated proteins	3985	3534	6238	4490
True positives	-	3084	3463	3057
False positives	-	450	2775	1433
False negatives	-	901	522	928
Sensitivity	-	0.77	0.87	0.77
Precision	-	0.88	0.56	0.68
F1 score	-	0.82	0.68	0.72

^a^Hori et al. [4]

The F1 score (0.82) for the comparison of Hotpep with Hori et al. [4] for the 16 fungal genomes is a little lower than the F1 score (0.86) for the annotation of the 12 bacterial genomes. However, the fungal genomes were all from basidiomycetes that are less represented in the CAZy database than carbohydrate-active enzymes from ascomycetes and thus may be more difficult to annotate. To assess this possibility we used previously published data [10] to calculate the F1 score for comparison of annotation of six ascomycete genomes by Hotpep and the CAZy database tools for annotation. The few disagreements between the methods were attributed mainly to differences in gene prediction rather than to differences in annotation [10]. In line with this notion, the F1 score for this dataset of ascomycete genes was 0.92 compared to only 0.82 for the annotation of basidiomycete genes in the present study. This finding suggests that the publicly available CAZy database may not yet account for the complete sequence variation in the carbohydrate-active enzyme families. E.g., the basidiomycete sequences may be underrepresented. This is in agreement with the ongoing addition of new sequences to the CAZy database [1]. A simple expansion of the LPMO enzyme families in the CAZy database by including previously unannotated, publicly available sequences led to the identification of the AA11 enzymes [9] and was shown to give a better representation of the sequence variation of the families, hereby making it possible to identify 31% more LPMOs in 39 fungal genomes [13]. The current version of Hotpep for annotation of carbohydrate-active enzymes include the expanded conserved peptide signatures for the AA9, AA10 and AA11 families. As expanded signatures become available for other families, they will be added to Hotpep.

Hotpep could principally be used for annotation of other enzymes than carbohydrate-active enzymes provided that sufficiently well curated sequence data bases are available.

## Conclusion

Hotpep is an easy to use tool that performs automatic annotation of carbohydrate-active enzymes with high success rate. The result of annotation with Hotpep is comparable to state-of-the-art semiautomatic annotation by experts [1, 4] and automatic annotation with HMMs [7]. Furthermore, Hotpep also provides a functional prediction of function directly from amino acid sequence.

A downloadable version of Hotpep is available as a stand-alone application that runs on the MS Windows operative system.

## Additional files

Additional file 1: Conserved Peptide Patterns for all Carbohydrate-Active Enzyme Families and CBMs. This file includes all conserved peptide patterns for all PPR groups and functional data for the enzymes in each group. (XLSX 2251 kb)

Additional file 2: Hotpep annotation of CBMs based on conserved peptides identified by PPR analysis. This file includes the results of Hotpep annotation of CBMs based on conserved peptides identified by PPR analysis with different parameters as indicated. (XLSX 15 kb)

Additional file 3: Hotpep functional prediction of 8812 experimentally characterized enzymes. This file includes experimental activity data from the CAZy database compared to Hotpep predictions for 8812 carbohydrate-active enzymes. (XLSX 235 kb)

Additional file 4: Comparison of Hotpep annotation from genomes and from predicted proteins. This file includes the results of Hotpep annotation of carbohydrate-active enzymes in seven fungal genomes and in the predicted proteins from the genomes. (XLSX 15 kb)

## Abbreviations

AA: Auxiliary activities
CBM: Carbohydrate binding module
CE: Carbohydrate esterases
GH: Glycoside hydrolases
GT: Glycosyl transferases
HMM: Hidden Markov Models
Hotpep: Homology to Peptide Pattern
LPMO: lytic polysaccharide monooxygenase
PL: Polysaccharide lyases
PPR: Peptide Pattern Recognition
